# Introduction

**DOI:** 10.1007/978-3-030-61315-0_1

**Published:** 2020-12-08

**Authors:** Colin Ray Anderson, Janneke Bruil, M. Jahi Chappell, Csilla Kiss, Michel Patrick Pimbert

**Affiliations:** 6 Centre for Agroecology, Water and Resilience, Coventry University, Wolston, UK; 7 Cultivate! Collective, Bennekom, The Netherlands; 8 Centre for Agroecology, Water and Resilience, Coventry University, Coventry, UK

**Keywords:** Agroecology, Transformation, Crisis, Social movements

## Abstract

In this introductory chapter, we introduce agroecology as an urgent alternative paradigm for food and farming in a time of growing ecological, economic and social crises. We briefly outline the role of food systems in these intersecting crises and introduce how agroecology is much more than a ‘technical fix’ that calls to tweak the existing system. It is rather a framework for transformation that can be adopted in pursuit of a more just and sustainable food system. The chapter describes the origin of the book and provides a roadmap to help the reader navigate the flow of the manuscript.

## Agroecology: An Idea for Urgent Times

In her recent analysis of the COVID-19 pandemic, Canadian journalist Naomi Klein ironically riffs off a famous phrase on crises by free-market economist Milton Friedman. When catastrophe hits, he noted, “the actions that are taken depend on the ideas that are lying around” (Klein 2020). In the context of current and imminent crises—from climate change and biodiversity loss to hunger, poverty and disease—it is clear that catastrophe is not only on our doorstep but has arrived for many peoples around the world. It is also clear that agroecology is not just an idea that is ‘lying around’ but one that has been teed up by visionary food producers, social movements and researchers. The time for agroecology is now.

Over the past five years, the theory and practice of agroecology have crystalized as an alternative paradigm and vision for food systems. Agroecology is an approach to agriculture and food systems that mimics nature, stresses the importance of local knowledge and participatory processes and prioritizes the agency and voice of food producers over corporations and other elite actors. As a traditional practice, its history stretches back millennia, whereas a more contemporary agroecology has been developed and articulated in scientific and social movement circles over the last century. Most recently, agroecology—practised by hundreds of millions of farmers around the globe—has become increasingly viewed as viable, necessary and politically possible as the limitations and destructiveness of ‘business as usual’ in agriculture have been laid bare.

But as a system, agroecology has powerful competition in the corporate actors who peddle high-tech, profit-centred ‘solutions’ that preserve an unjust and unsustainable food system and agroeclogy remains marginal, its potential effectively sabotaged by the political interests that continue to embolden the high-input industrial model. The battle for the future of food and farming is intensifying with the growing sense of urgency over our intermeshed ecological and social crises.

There is now much evidence to show that our socio-economic systems are catastrophically undermining the function of natural systems. The Intergovernmental Panel on Climate Change (IPCC) (2019) notes that between 2007 and 2016 some 23% of total anthropogenic greenhouse gas emissions came from unsustainable practices in agriculture, forestry and other land-use activities. Other major reports have drawn attention to convergent crises such as accelerating extinction rates (IPBES 2019), looming water shortages for five billion people (World Water Assessment Programme (WWAP) 2019), UNESCO rising world hunger (FAO 2019), dangerous degradation and pollution of land and soil, mounting resource depletion and a rise in levels of air pollution resulting in disease and health-related death (Health Effects Institute 2018). And, most recently, the COVID-19 crisis has revealed the vulnerability that arises from a just-in-time, centralized industrial food system (Wallace et al. 2020).

In fact, the pandemic has revealed how industrial agriculture contributes to the rise and spread of deadly pathogens by pushing agriculture and extraction further into the forest and by creating densely crowded genetically homogenous domestic livestock populations that are breeding grounds for the emergence of zoonotic viruses (Wallace 2016; Wallace et al. 2020). ‘Industrial food’, as a system, both spawns large-scale ecological, social and economic problems and reduces the capacity or resiliency of farmers and communities to cope with change. Major shifts are needed, not tweaks to the failing system we have.

**Fig. 1.1:**
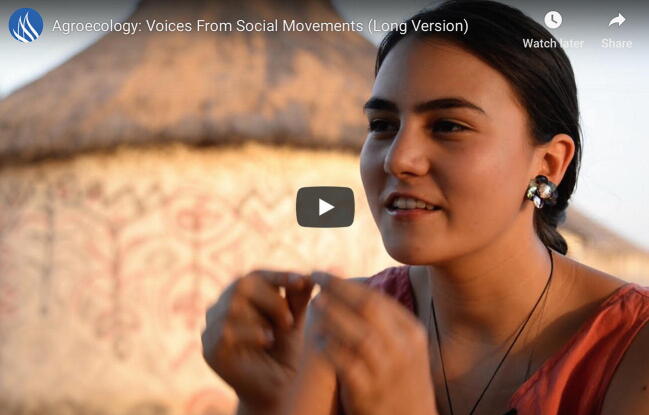
*The film Agroecology*: Voices from *Social* *Movements* exemplifies the book’s primary theme: the struggle to advance agroecology as an alternative to the dominant food regime.View video here: https://www.agroecologynow.com/video/ag/ (*Photo credit: Authors*)

Despite significant underfunding and lack of research (see Chap. 10.1007/978-3-030-61315-0_5), evidence on the multifunctional benefits of agroecology are growing (summarized in Chap. 10.1007/978-3-030-61315-0_2). In contrast, agroecology represents a system that works with nature instead of against it and offers an approach to food production that boosts biodiversity, creates ecological resilience, improves soils, cools the planet and reduces energy and resource use. It has been shown to be highly productive, to provide highly diverse dietary offerings and to support the process of community building and women’s empowerment (Fig. 1.1).

The agroecology that we embrace in this book emerges not only as an alternative to the oft-critiqued industrial and corporate food system, however. It must also be part of the effort to counter racial capitalism, patriarchy and other forms of structural violence and oppression. Although anti-racism, indigenous cosmovision, decolonization and feminism are often found only in the radical margins of the agroecology canon, it is in these traditions that the transformative potential of agroecology can be deepened. Movements from Black Lives Matter to the World March of Women offer potential lessons and allies for agroecology. So do on-the-ground experiments with equity and radical democracy, such as those taking place in the autonomous region of Rojava in Syria, and the work of action researchers exploring decoloniality, feminist political ecology, queer ecology, critical physical geography and beyond.

In this context, a transformative agroecology can be imagined as one manifestation of a global struggle for emancipation—achievable through solidarities, ally-ship and strategic action. Thus, while food systems are this book’s focus, we make connections throughout to the intersection with wider struggles against oppressions and call for the field and practitioners of agroecology to integrate further with these wider movements for change.

A deeply politicized and collectivized practice of building agroecology from the bottom up is, we argue, the essential *basis* for transformation in food systems. We believe that this will happen only when the dominant regime is itself transformed to enable agroecology as *an objective of transformation*. The dialectical process is central to the aim. We take an agency-centric approach (see, e.g., the discussion of agency in HLPE (2019), working alongside our allies from many walks of life, in facing this challenge to the hierarchies and assumptions of the dominant regime. Our approach identifies the need for substantial shifts in governance and power. If agroecology is indeed a good idea that is lying around, it is time to map out how we can seize the moment for the transition to a more just and sustainable food system, and society.

## The Origins and Purpose of the Book

This book is the result of a research collaboration that started in 2018 with a literature review and case-study development for the Food and Agriculture Organization of the United Nations (FAO). Our eclectic group of activist scholars set out to understand how to amplify agroecology while moving towards just, sustainable food systems. We analysed academic and non-academic literature to better understand the dynamics involved in agroecology transitions, the opportunities and obstacles, and the role of governance and power. We also invited a small number of people with practical and/or academic experience in agroecology to submit new case studies as a way of deepening our understanding of particular aspects of the field.

At the end of 2018, an initial version of this research was presented at a multiday workshop in Rome involving academics, social-movement leaders and FAO staff from around the world. We continue to be grateful for their suggestions for improvements. Since then, FAO has used our report internally as part of its global policy process to support the scaling up of agroecology.

In 2019, we published snippets of our findings, for example in an article in the journal *Sustainability* and as a ‘backgrounder’ (Anderson et al. 2019). Following that, we were told by friends and colleagues that a publicly accessible version of the full research paper would be very timely. They encouraged us to use the opportunity to publicize the idea of a transformative approach to agroecology more widely. We are, after all, at a crucial moment in this effort, as agroecology gains traction not only with FAO but also with national governments, social movements and other actors—with the associated risks and opportunities. The idea of an open access publication emerged. Palgrave Macmillan agreed to publish an updated version of our work: the result is this book.

In it, we seek to provide insights into approaches to agroecology, based on core principles adapted to place and context rather than proscriptive rules. We articulate agroecology as an ongoing *process* of food-system transformation, supported by a set of underlying *values* based on ecological principles and social justice, and honouring the *agency* of food producers and the important role of social movements in transformational change. Thus, while our aim is to understand and support large-scale transformational change, our approach is to focus on the tangible changes that are possible when working from the bottom up in communities and social movements. This requires a simultaneous process of strengthening and building agroecology as a radical alternative while also deconstructing the dominant corporate food regime that lock in unsustainable and unjust food systems.

Ultimately, this book aims to serve, directly or indirectly, agroecologists—particularly organizations and networks of agricultural producers, and especially women. Much of the thinking that went into it has been inspired by what we have learned from them. We hope the combination of a theoretical and analytical framework with more empirical analyses (including case studies) will offer intellectual and practical inspiration to academics and students keen to understand how territorial efforts may be connected to system-wide transformations.

As we have noted, our findings will also speak to people in other political movements—from climate and environmental justice to anti-racism, de-growth and feminism. We believe that the insights are relevant too to policy-makers, journalists and other advocates of healthier, more sustainable and accessible food and agriculture systems.

## A Roadmap to the Book

In Part I, we elaborate on the history, meaning and multiple ecological, economic and social benefits of agroecology. We then introduce the notion of a transformative agroecology rooted in the tradition of political ecology adopted in this book. To better conceptualize the process of transformation, we use the multi-level perspective—an influential framework for analysing sustainability transitions across space and time (Geels 2011; Geels and Kemp 2007). With this approach, we show how agroecology—which emphasizes the agency of people—sits within a dominant regime that operates through deep ‘landscape’ level processes of capitalism, racism, patriarchy and colonialism. It is in the interface and conflict between these two paradigms that transformation—spurred by collective action, shifts in governance and building of countervailing power—can occur.

In Part II, we introduce the idea of ‘domains of transformation’, which we flesh out as discrete conceptual areas within which the dominant regime poses barriers to the development of agroecology. On the other hand, it is also within each of these domains that proponents of agroecology are taking collective to advance the transformative project at the heart of agroecology. Thus, the domains represent discrete but deeply interconnected areas where the regime and agroecology collide and where further interventions are required to enable agroecology transformations. Synthesizing the literature and bringing in case studies and vignettes from our research and networks, we present six such domains: rights and access to nature (Chap. 10.1007/978-3-030-61315-0_4); knowledge and culture (Chap. 10.1007/978-3-030-61315-0_5); systems of economic exchange (Chap. 10.1007/978-3-030-61315-0_6); networks (Chap. 10.1007/978-3-030-61315-0_7); equity (Chap. 10.1007/978-3-030-61315-0_8) and discourse (Chap. 10.1007/978-3-030-61315-0_9). However, as will be demonstrated, efforts in one domain alone are insufficient and it is a holistic and integrated approach across all of these domains where the greatest potential for agroecology transformations manifests.

Finally, in Part III, we drill down on issues of governance, power and control across all six domains to find the fundamental drivers of transformation through agroecology. We have identified six distinct ways in which different governance interventions (such as new state policies, the building of new ‘nested markets’, and the actions of civil society networks) affect the dynamics between the dominant food system and emergent agroecological alternatives. When top-down technocratic approaches in governance shift towards bottom-up distributed ones, agroecology is enabled in all the domains, and ultimately, as the changes in each domain overlap, they will synergize towards a system-wide shift.
